# Exploring Utilization and Establishing Reference Intervals for the Apolipoprotein B Test in the Korean Population

**DOI:** 10.3390/diagnostics13203194

**Published:** 2023-10-12

**Authors:** Rihwa Choi, Sang Gon Lee, Eun Hee Lee

**Affiliations:** 1 Department of Laboratory Medicine, Green Cross Laboratories, Yongin 16924, Republic of Korea; pirate0720@naver.com; 2 Department of Laboratory Medicine and Genetics, Samsung Medical Center, Sungkyunkwan University School of Medicine, Seoul 06351, Republic of Korea; 3 Green Cross Laboratories, Yongin 16924, Republic of Korea

**Keywords:** lipid, apolipoprotein B, ApoB, dyslipidemia, reference interval, Korean

## Abstract

We investigated the reference intervals for Apolipoprotein B (ApoB), a valuable biomarker for cardiovascular diseases, in Korean adults who had undergone health check-ups and showed normal lipid levels under traditional lipid tests, including total cholesterol, triglyceride, and high-density lipoprotein cholesterol, along with ApoB. We compared the findings with different cutoffs for ApoB from international clinical guidelines. Among a total of 264,105 traditional lipid test sets, only 464 (0.2%) included ApoB tests, indicating underutilization of this test in health check-up clinics in Korea. From these 464 samples, 334 ApoB results (164 men and 170 women) with normal traditional lipid test results were used to establish reference intervals. Using the parametric method (mean ± 2 SD), the reference intervals ranged from 46 to 134 mg/dL for men and 49 to 129 mg/dL for women. Employing the non-parametric method (central 95th percentile value), the reference intervals ranged from 50 to 131 mg/dL for men and 51 to 127 mg/dL for women. The prevalence of high ApoB did not significantly differ by sex when considering the established reference intervals for each sex and the cutoffs recommended by international clinical guidelines. This study enhances knowledge on ApoB reference intervals in the Korean population, and it will in aid test result interpretation for clinicians and laboratories.

## 1. Introduction

Cardiovascular diseases (CVDs) remain a major global health burden, contributing to a significant number of deaths worldwide [1,2]. The early identification and accurate assessment of cardiovascular risk are crucial for implementing appropriate preventive measures and optimizing patient outcomes [3,4,5]. Traditional lipid parameters, including total cholesterol (TC), triglycerides (TG), low-density lipoprotein cholesterol (LDL-C), and high-density lipoprotein cholesterol (HDL-C), have been widely utilized as indicators of CVD risk for a considerable period [3,4,6,7,8,9]. However, these measurements fail to capture the full spectrum of atherogenic lipoproteins and may not accurately reflect the actual burden of atherogenic particles in an individual [7,8]. Non-HDL-C (calculated from TC-HDL-C) and apolipoprotein B (ApoB) are additional lipid parameters implicated in the development and progression of CVDs [3,4,10]. Among the lipoproteins, ApoB, the main structural protein of LDL and very-low-density lipoprotein (VLDL) particles, plays a central role in lipid metabolism and serves as the ligand for cellular receptors that facilitate the uptake of lipoproteins by various tissues, including arterial walls [4,7,8]. The concentration of ApoB reflects the number of atherogenic particles in circulation, making it a valuable marker for assessing cardiovascular risk [3,4,5,8,11].

In recent years, advancements in laboratory techniques have facilitated the development of reliable and standardized assays for measuring ApoB levels [11,12,13]. These assays offer clinicians a practical tool for evaluating a patient’s cardiovascular risk profile, aiding in personalized treatment strategies and monitoring therapeutic interventions [3,5,7,8,14]. However, the status of assay standardization for measuring ApoB has not been fully achieved, limiting its wide application, and caution should be exercised when interpreting results using reference intervals [12,15].

To effectively utilize ApoB measurements in clinical practice, it is essential to establish reliable reference intervals that provide a baseline for interpretation and decision-making [16]. Reference intervals, also known as normal ranges, define the range of values observed in a healthy population for a specific laboratory parameter [17,18]. These intervals serve as benchmarks against which individual test results can be compared, aiding in the diagnosis, monitoring, and management of various medical conditions [17,19,20]. Accurate and reliable reference intervals for ApoB are crucial for clinicians to interpret test results and make informed decisions regarding cardiovascular risk assessment and treatment strategies [20]. In clinical laboratories, reference intervals can either be transferred from various sources, followed by verification studies, or they can be established using a laboratory’s own population [18,19,20].

Recent international guidelines on dyslipidemia, such as those issued by the Ameri-can Heart Association (AHA), American College of Cardiology (ACC), Multisociety (MS), European Society of Cardiology (ESC), European Atherosclerosis Society (EAS), and Canadian Cardiovascular Society (CCS), emphasize the role of ApoB as a valuable biomarker for cardiovascular risk assessment [3,4,5]. They highlight its superiority over traditional lipid parameters in certain populations and provide target levels for ApoB [3,4,5]. Various clinical guidelines offer distinct cutoff values for ApoB to identify individuals at risk and guide decision-making in management processes [3,4,5]. However, according to the current Korean guidelines for dyslipidemia, ApoB is recommended as an additional biomarker in specific clinical situations, such as diabetes and familial hypercholesterolemia, without specific cutoff values [21,22]. Clinicians should understand the use of specific cutoff values for ApoB with caution because the analytical method standardization for ApoB is not perfect [12,23]. Although reference materials have been developed, significant differences have still been reported among the analytical methods [11,15,23]. Moreover, there are limited data available for reference intervals for ApoB in the Korean adult population [14,19,20,21,24,25,26,27]. Previous studies conducted on Korean populations have used different analytical platforms and primarily focused on risk assessments for cardiovascular disease rather than establishing reference intervals for ApoB [14,19,20,21,24,25,26,27,28].

Therefore, in this study, we investigated reference intervals for ApoB in Korean adults who underwent lipid profile tests, along with ApoB, during health check-ups. We employed different methods in this investigation that have been widely used in clinical laboratories [18,19,20]. Furthermore, we compared these reference intervals with previously reported results and the cutoffs recommended by international clinical guidelines [3,4,5]. In this paper, we share our approaches to investigating and reviewing the reference intervals for ApoB in the Korean population. This study can provide implications for professionals working in clinical laboratories in applying reference intervals for ApoB and for physicians to understand and use caution in interpreting ApoB results.

## 2. Materials and Methods

### 2.1. Source of Reference Intervals

At Green Cross Laboratories, traditional lipid tests including TC, TG, and ApoB were performed using automated analyzers [2,29]. The analytical method for ApoB was per-formed using a Tina-quant Apolipoprotein B version 2 reagent kit (Roche, Mannheim, Germany) traceable to the IFCC reference material SP3-07 on automated c702 analyzers (Roche, Germany) [2]. The reference intervals for ApoB were transferred from the manufacturer’s information, after a verification study, following its implementation in the laboratory [19,20]. At the time of implementing the ApoB test, the reference intervals provided by the manufacturer were 66 to 133 mg/dL for men and 60 to 117 mg/dL for women, and the manufacturer claimed that these values were obtained using serum samples from healthy subjects. However, there was no detailed information regarding how those ranges were established by the manufacturer.

In December 2022, the manufacturer updated the reference intervals in their reagent instructions for use without any analytical method changes (i.e., no change in the reagent composition, etc.). They stated that the updated reference intervals were derived from data from Contois et al., which included results from the Framingham Offspring Study published in 1996 [17]. The updated reference intervals provided by the manufacturer were 66 to 144 mg/dL for men and 60 to 141 mg/dL for women. These intervals were based on the central 90th percentile values of the data from Contois et al., resulting in an expansion of the upper limits of the reference intervals [17]. As there were no changes in the analytical methods since their implementation, we conducted a retrospective review of lipid test results, including ApoB tests, performed between 2020 and 2022 at Green Cross Laboratories through the laboratory information system.

To perform a comprehensive analysis and the investigate reference intervals for the Korean population, we established the reference intervals using an indirect method [18]. The reference intervals were established in accordance with the Clinical and Laboratory Standards Institute (CLSI) guidelines and previous literature [18,19]. This involved a retrospective review of laboratory test results from Korean adults who underwent ApoB tests and traditional lipid profile tests (TC, TG, and HDL-C) for health check-up purposes between 1 January 2020 and 31 December 2022 through the laboratory information system of Green Cross Laboratories [18,30,31,32]. All data were anonymized before statistical analysis. Because the aim of this study was to establish reference intervals for ApoB using data from individuals, repeated test results for the same individuals were excluded. After exclusion, laboratory test results were included only if they met the following criteria: TC < 240 mg/dL (non-hypercholesterolemia), TG < 200 mg/dL (non-hypertriglyceridemia), non-HDL-C < 190 mg/dL, and calculated LDL-C < 160 mg/dL using all three equations, including the Friedewald equation, Martin/Hopkins equation, and Sampson/NIH equation, according to the Criteria for the National Cholesterol Education Program (NCEP) Adult Treatment Panel III (ATP III) criteria [2,21,22,30,33,34,35,36]. The study population used to establish the reference intervals consisted of Koreans who had visited local clinics and hospitals for routine health check-ups and did not exhibit traditional lipid test abnormalities based on the NCEP ATP III criteria, which define subjects without dyslipidemia. For example, the cutoff value of 240 mg/dL for TC was derived from the NCEP ATP III criteria, and it is a globally accepted threshold for identifying individuals with high TC levels. It is worth noting that this cutoff value of 240 mg/dL was also used to assess the prevalence of hypercholesterolemia in both the National Health and Nutrition Examination Survey (NHANES) in the United States by the Centers for Disease Control and Prevention (CDC) and the Korea National Health and Nutrition Examination Survey (KNHANES).

The accuracy of a traditional lipid profile test is ensured through participation in accuracy-based external quality assurance programs, such as those provided by the Centers for Disease Control in the United States (Lipids Standardization Program); the College of American Pathologists (ABL surveys); and the Korean External Quality Assessment Scheme [37].

### 2.2. Investigation of Reference Intervals

The reference intervals investigation involved both parametric (mean ± 2 SD, reference interval (1)) and non-parametric (central 95th percentile; 2.5 to 97.5, reference interval (2)) methods. The Kolmogorov–Smirnov test was utilized to assess the normality of the ApoB results. The reference intervals were determined separately for each sex. We compared the established reference intervals with the manufacturer’s information, previously reported ranges in the Korean population, and cutoff values recommended by current international clinical guidelines for Western populations. The prevalence of patients with high ApoB values, according to the different cutoff values from the upper limits of the reference intervals and from the clinical guidelines, was also investigated by sex using chi-squared tests. The statistical analysis was performed using MedCalc statistical software version 20.116 (MedCalc Software Ltd., Ostend, Belgium; https://www.medcalc.org; 2023, accessed on 25 July 2023). *p*-values were considered significant at the 0.05 level.

## 3. Results

**Figure 1:**
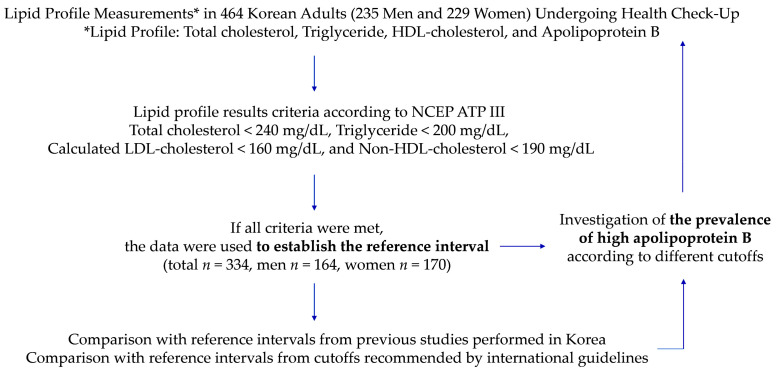
Study scheme.

During the three-year study period, a total of 264,105 traditional lipid test sets (TC, TG, and HDL-C) were conducted, with 59,991 test sets in 2020, 90,042 test sets in 2021, and 114,072 test sets in 2022. Among these, only 464 test sets (0.2%) included simultaneous measurements of ApoB, indicating its underutilization in Korean adults during health check-ups. A further analysis included 334 test sets from 164 men and 170 women without hypercholesterolemia, hypertriglyceridemia, increased non-HDL-C, and increased calculated LDL-C for the establishment of the reference intervals. The study scheme is summarized in Figure 1, and the baseline characteristics of the study subjects are presented in Table 1. The median age of the subjects was 59.6 years (interquartile range: 47.4 to 67.3). The data were not normally distributed, except for ApoB, and they were presented as medians and interquartile ranges. The mean ApoB value was 89.5 mg/dL (SD 20.9) for the total subjects, 90.2 mg/dL (SD 22.0) for men, and 88.9 mg/dL (SD 19.9) for women. A Mann–Whitney U test was used to compare the age and lipid results by sex, and it revealed no significant differences in the parameters, except for the HDL-C levels (*p* = 0.0006 for HDL-C and *p* ≥ 0.05 for the others). A chi-squared test was used to compare the ApoB levels by sex, and it revealed no statistically significant differences (*p* ≥ 0.05).

The distribution of the ApoB levels by sex is summarized in Table 2. The ApoB levels were found to be normally distributed in the total subjects as well as in each sex. The reference intervals for ApoB were defined as 46 to 134 mg/dL for men and 49 to 129 mg/dL for women based on the mean ± 2 SD (reference interval 1). Alternatively, using the central 95th percentile value, the reference intervals for ApoB could also be 50 to 131 mg/dL for men and 51 to 127 mg/dL for women (reference interval 2). It was observed that the reference intervals established using the mean ± 2 SD were slightly wider than those established using the central 95th percentile values.

**Figure 2:**
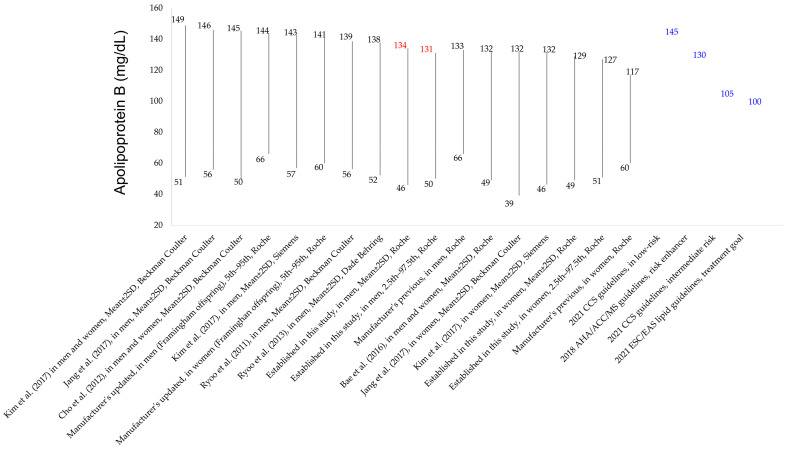
Reference intervals and ranges of apolipoprotein B (ApoB) levels investigated in Korean populations using different statistical approaches, analytical methods, and cutoff values provided by international guidelines for dyslipidemia. The upper limits of the reference intervals obtained from this study’s population are represented by the numbers in red while the cutoff values provided by the international guidelines are shown in blue. The *x*-axis indicates the sources of the reference intervals and ApoB ranges while the *y*-axis represents the ApoB levels in mg/dL. Previous studies on ApoB in the Korean population, including those by Kim et al. (2017) [29], Jang et al. (2017) [27], Cho et al. (2012) [26], Kim et al. (2017) [28], Ryoo et al. (2011) [21], Ryoo et al. (2013) [20], and Bae et al. [25], employed various analytical methods. Data were presented in descending order of the upper limits.

The established reference intervals were compared with previous ApoB results from the literature and current international guidelines for dyslipidemia (Figure 2). Although the previous studies conducted on Korean adults did not specifically focus on establishing reference intervals for ApoB, they presented ApoB results from healthy populations who underwent ApoB measurements for health check-ups. These previous studies measured ApoB using different analytical methods (Beckman Coulter or Siemens) compared to the present study population (Roche).

**Figure 3:**
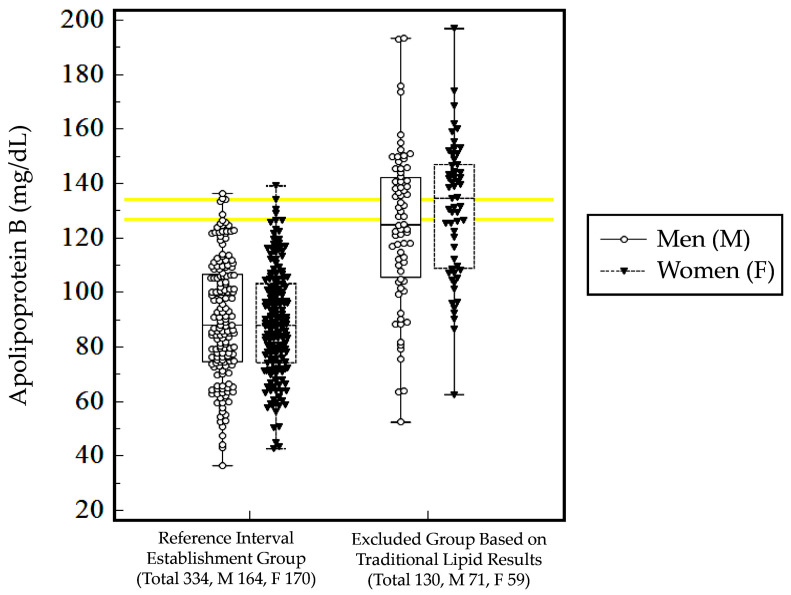
Apolipoprotein B levels observed in the 334 Korean adults for the establishment of the reference intervals and the 130 Korean adults excluded from the reference interval establishment. The yellow horizontal lines represent the highest and lowest upper limits of the established reference intervals in this study’s population. The upper yellow horizontal line represents 134 mg/dL for the men, which was determined from the mean (±2 SD) value, while the lower yellow horizontal line represents 127 mg/dL for the women, which was derived from the central 95th percentile value.

The mean (± SD) ApoB results for the 334 Korean adults (89.5 ± 20.9 mg/dL) used for establishing the reference intervals were significantly different from those of the other 130 Korean adults (127.0 ± 27.0 mg/dL) who were excluded from the reference interval establishment due to their traditional lipid result criteria (*p* < 0.0001, Figure 3). There were no statistical differences in the ApoB levels between the men and women in the 130 Korean adults who were excluded from the reference interval establishment (*p* ≥ 0.05).

**Figure 4:**
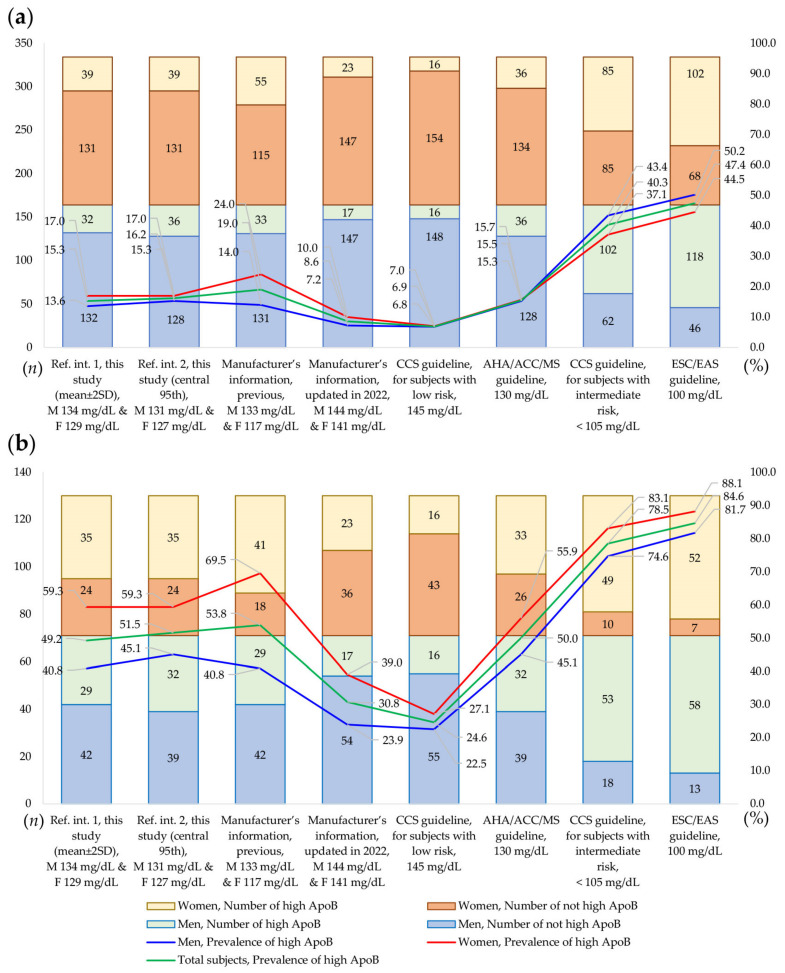
Prevalence of high apolipoprotein B (ApoB) levels using different cutoff values (upper limits of the reference intervals) in (**a**) the 334 Korean adults who underwent health check-ups and had traditional lipid test results within the NCEP ATP III criteria and (**b**) the 130 Korean adults who were excluded from the population for reference interval establishment due to their traditional lipid result criteria. The *x*-axis represents the sources and cutoff values for the ApoB levels. The *y*-axis on the left represents the number of subjects, and the *y*-axis on the right represents the percentages (%) of study subjects with high ApoB levels. “M” represents men and “F” represents women.

The prevalence of subjects with high ApoB levels was compared using the different upper limits of the reference intervals and cutoff values from a total of 464 subjects (Figure 4). Among the 334 Korean adults who underwent health check-ups with traditional lipid test results that were within the NCEP ATP III criteria, the prevalence of high ApoB levels ranged from 6.9% to 47.4%, depending on the cutoff value used. For the 130 Korean adults excluded from the reference interval establishment group, the prevalence of high ApoB levels ranged from 22.5% to 88.1%, depending on the cutoff value used. There was a significant difference in the prevalence of high ApoB levels between men and women when using the reference interval transferred from the manufacturer’s previous information (133 mg/dL for men and 141 mg/dL for women, *p* = 0.0062). However, the prevalence of high ApoB levels did not show a significant difference by sex with the application of other cutoffs (*p* ≥ 0.05). Notably, the prevalence of high ApoB levels was nearly the same (within 0.4% difference) when using the cutoff value recommended for subjects with low risk by the 2021 CCS guidelines (<145 mg/dL) and the cutoff value recommended by the 2018 AHA/ACC/MS guidelines (<130 mg/dL).

## 4. Discussion

In this study, we evaluated reference intervals for ApoB levels using an indirect method on Korean adults who underwent health check-ups and had traditional lipid test results that excluded abnormalities based on the NCEP ATP III criteria. The results showed that ApoB tests were underutilized in health check-up clinics, accounting for only 0.2% of traditional lipid tests, and this was consistent with previous findings in Korean patients visiting local clinics and hospitals (2.0%) [2]. The appropriate utilization of ApoB testing could be achieved by providing relevant information about its use and interpretation, along with applicable tools, and aligning it with national guidelines for education and practical application [2,16]. There is still uncertainty regarding whether the increased accuracy of ApoB testing justifies a paradigm shift from the traditional lipid profile tests used in clinical practice [38]. The cost–benefit analysis of incorporating an ApoB test into a traditional lipid panel for the Korean population requires further study and thorough evaluation to enhance its clinical utility [39,40].

There are two primary methods used to establish reference intervals: direct methods using prospectively collected data from well-defined healthy subjects and indirect methods using retrospective data analyses [18,19]. Each method has its own set of advantages and disadvantages [18]. The direct method, which involves well-defined healthy subjects, can provide the most ideal reference intervals [18,19]. However, it is widely acknowledged that establishing a reference interval using the direct method can be exceptionally challenging for most clinical laboratories due to limited resources [18,19]. In contrast, the indirect method can be easily conducted in clinical laboratories by retrospectively analyzing data, but it is susceptible to selection bias [18,19]. A reference interval is typically defined as the range within which values from most healthy subjects fall [19]. These values can be obtained through descriptive data analysis using means and 2 SD or central 95th percentile values [19]. In contrast, a cutoff value, a term often used in conjunction with reference intervals, is established to effectively differentiate between healthy subjects and those with specific conditions (e.g., hypercholesterolemia) [19]. Determining a cutoff value involves a receiver operating curve (ROC) analysis, which takes into account sensitivity, specificity, and predictive values [19]. The choice of a cutoff value depends on the analyte’s objective, whether it is to assess risk, screen, diagnose, confirm, predict a prognosis, monitor disease, or evaluate outcomes [19]. In this study, we employed indirect methods to establish the reference intervals, and we employed descriptive statistical methods, including both parametric and non-parametric analyses.

We presented a practical approach for establishing reference intervals, accompanied by a review of the current guidelines, highlighting their significance as a crucial step for clinical laboratories. In clinical laboratories with limited resources, a critical step is to assess the existing literature to understand reference intervals in a specific population of interest [19]. We evaluated previous studies conducted on Korean populations to determine if the reference intervals established in our study aligned with current knowledge. The establishment of reference intervals for ApoB involves several critical considerations [11,20]. Firstly, a representative and diverse healthy population must be selected, encompassing individuals of different ages, ethnicities, and lifestyles [20]. The use of a standardized and validated ApoB assay is essential for ensuring the accuracy and comparability of the results across different laboratories and populations [11,15,24,41]. Standardization plays a pivotal role in ensuring the uniformity, comparability, and accuracy of laboratory test results [11,12,15,24]. The complex nature of ApoB metabolism and the diverse assays used for its measurement have posed unique challenges [12]. Various analytical methods, including immunoturbidimetry, immunonephelometry, and enzyme-linked immuno-sorbent assays, have been employed for ApoB measurements [9,11,12]. However, significant variability has been observed in ApoB results obtained from different methods, making it challenging to establish consistent reference intervals and clinical decision thresholds [11,12,15,24]. Standardized ApoB measurements enhance the accuracy and precision of cardiovascular risk assessment by providing more reliable estimates of atherogenic particle burden, and in turn, they facilitate better risk stratification, patient management, and assessment of therapeutic interventions [11,41,42].

In the present study, reference intervals for ApoB levels were established using both parametric (mean ± SD) and non-parametric methods (central 95th percentile value), with the reference intervals using the parametric method being slightly wider than those that used the non-parametric method [18,21]. In comparison with the ApoB test results reported in previous studies on healthy Korean subjects using different analytical platforms, different ranges for ApoB levels were observed. The ApoB results from healthy subjects measured by automated immunoturbidimetric assays manufactured by Beckman Coulter showed higher upper limits than the reference intervals from this study [21,26,27,28]. The cutoff value for ApoB recommended by the 2021 CCS guideline (<145 mg/dL for subjects with low risk) appeared similar to the upper limits of the ApoB results from the Korean population using Beckman Coulter assays and the manufacturer’s updated information in 2022. The cutoff values by the 2018 AHA/ACC/MS guidelines (130 mg/dL) appeared to be similar to the upper limits of the reference intervals established in this study using the Roche assay platform. These results were consistent with those of previous studies regarding ApoB levels using different assay platforms, which reported higher ApoB levels measured by Beckman Coulter than those established by Roche platforms in comparability studies using the reference material SP3-07 [43,44]. The prevalence of high ApoB levels showed significant differences depending on the cutoff values used in this study. Significant differences in the prevalence of high ApoB levels by sex were observed only when the cutoff value provided by the manufacturer’s previous information was used (133 mg/dL for men and 117 mg/dL for women). These cutoff values (133 mg/dL for men and 117 mg/dL for women) were the same levels used in the NHANES 2015-2016 study conducted by the United States CDC, which provided reference intervals using the Roche manufacturer’s data [45]. An important note is that in December 2022, the manufacturer Roche raised the upper limit of the reference interval for women from 117 mg/dL to 141 mg/dL without any changes in the analytical method. This new upper limit of 141 mg/dL was even higher than the upper limit of the reference interval for women established in our study, which was 131 mg/dL. This new value of 141 mg/dL significantly exceeded the cutoff value of 130 mg/dL, as recommended by the AHA/ACC/Multi-society guidelines for defining individuals with risk-enhancing factors for cardiovascular diseases [3]. The upper limits of the reference intervals we established in our study closely aligned with those in the AHA/ACC/Multi-society guidelines. In the present study, there were no significant differences in the prevalence levels of high ApoB measurements using the other cutoff values. These results might have been due to the specimens having ApoB results ranging from 117 mg/dL to 127 mg/dL for women (7.0%) and from 130 mg/dL to 145 mg/dL for men (8.5%). When using the cutoff values recommended by the 2021 CCS guidelines for subjects with intermediate risk (105 mg/dL) and those in the 2021 ESC/EAS guidelines (100 mg/dL), the prevalence of high ApoB increased to approximately half (50.2%) in Korean men undergoing health check-ups, at maximum. Lipid metabolism and the levels of lipid tests can be influenced by various factors, including genetic factors, lifestyle, access to medical care, and government health policies [2]. As a result, lipid test data may vary somewhat when compared to data from other populations or they may not be found in the existing literature. For example, a recent study reported that age-adjusted mean TC levels in Korean women were lower than those in women from the United States, but the age-adjusted mean levels of LDL-C and HDL-C were comparable between the two groups [2]. According to the dyslipidemia fact sheet in Korea, which is based on KNHANES data, the crude prevalence of hypercholesterolemia in 2020 was 24% [46]. In the present study, the proportion of the excluded group based on the traditional lipid test results was 28.0% (130/464), which was comparable to the prevalence of hypercholesterolemia in the Korean population. Future studies are needed to identify the best cutoff values associated with clinical risks and outcomes for dyslipidemia and cardiovascular disease using a large number of study subjects with detailed clinical information, along with standardized assays.

The limitation of this study was the absence of clinical information associated with dyslipidemia and cardiovascular disease risk, such as family history, lifestyle, comorbidities, medications, etc. [3,4,5,21]. There is a possibility that excluding healthy individuals with elevated cholesterol levels detected during routine preventative visits may have affected the reference intervals. However, considering that dyslipidemia is typically asymptomatic and the NCEP ATP III criteria are globally recognized for defining dyslipidemia, this study still holds value as an example of how to explore reference intervals in clinical laboratories with limited resources [2,21,22,30,33,34,35,36]. In the present study, repeated measurements for the same individuals were excluded because the primary objective was to assess the reference intervals. Possible intraindividual variability might have influenced the ApoB level results. There may have been variations in medication use within the cohort, and it is highly probable that selection bias existed, considering the infrequent nature of the tests. It is important to note that due to the exclusion of a substantial number of ApoB test results, potential selection bias should be considered when interpreting the study’s findings. However, the strength of this study lies in its practical approach, utilizing a method commonly used in clinical laboratories to indirectly investigate and establish reference intervals [18,21]. The results of this study may be generalizable to clinical laboratories measuring ApoB using automated analytical assays from Roche and using specimens from Korean adults undergoing health check-ups. Future studies are needed to identify the best cutoff values for ApoB in the Korean population, considering different analytical methods and appropriate guidelines for interpreting test results specific to the Korean population.

## 5. Conclusions

In conclusion, we investigated the reference intervals of ApoB levels in Korean adults who underwent routine health check-ups and had normal traditional lipid levels. The ApoB test in Korean adults visiting health check-up clinics was found to be underutilized. In this study, we provided a practical method for establishing reference intervals in clinical laboratories, and the established reference intervals were closer to the cutoff values defining high ApoB levels that are recommended by international clinical guidelines than they were to the reference intervals provided by the manufacturer’s information. The prevalence of high ApoB levels in Korean adults undergoing health check-ups ranged from 6.9% to 47.4% depending on the cutoff value used, highlighting the importance of understanding and applying appropriate reference intervals and cutoff values in managing dyslipidemia and cardiovascular diseases. This study expands our knowledge about ApoB test utilization and the prevalence changes according to the cutoff value used. Future studies should validate the clinical implications of each cutoff value, including assessing accuracy and performance with detailed clinical information on dyslipidemia and cardiovascular diseases, to improve patient care.

## Figures and Tables

**Table 1:** Baseline characteristics of the 334 Korean subjects.

Characteristics	Total (*n* = 334)	Men (*n* = 164)	Women (*n* = 170)
Age, year, median	59.6 (47.4 to 67.3)	57.0 (43.8 to 68.8)	59.8 (48.1 to 64.7)
Total cholesterol, mg/dL	182.0 (159.0 to 205.0)	179.5 (151.5 to 205.0)	184.5 (166.0 to 205.0)
Triglyceride, mg/dL	95.0 (70.0 to 124.0)	102.0 (71.5 to 125.0)	87.5 (67.0 to 121.0)
HDL-C, mg/dL	57.0 (48.0 to 70.0)	55.5 (45.0 to 65.0)	61.5 (50.0 to 74.0)
Non-HDL-C, mg/dL	121.0 (99.0 to 147.0)	121.0 (96.5 to 152.5)	121.0 (100.0 to 145.0)
Cal.LDL-C, Friedewald equation, mg/dL [36]	103.0 (80.0 to 125.0)	101.0 (76.5 to 126.5)	106.0 (82.0 to 123.0)
Cal.LDL-C, Martin/Hopkins equation, mg/dL [35]	104.0 (82.0 to 124.0)	101.0 (78.0 to 129.5)	104.5 (84.0 to 122.0)
Cal.LDL-C, Sampson/NIH equation, mg/dL [34]	106.0 (83.0 to 126.0)	103.0 (78.5 to 130.0)	107.0 (85.0 to 125.0)
Apolipoprotein B, mg/dL	88.1 (74.4 to 105.3)	88.1 (74.7 to 106.7)	88.0 (74.2 to 103.2)

The data are presented as medians and interquartile ranges. Abbreviations: Cal, calculated.

**Table 2:** Distribution of apolipoprotein B levels in the 334 Korean subjects (mg/dL).

Sex	Min	2.5	5	10	25	50	75	90	95	97.5	Max	Mean	SD
Total	36.4	50.7	57.7	63.0	74.4	88.1	105.3	119.2	124.5	128.7	139.2	89.5	20.9
Men	36.4	49.5	55.0	62.7	74.7	88.1	106.7	122.0	125.0	130.5	136.4	90.2	22.0
Women	42.7	50.8	58.4	63.8	74.2	88.0	103.2	116.4	122.6	127.2	139.2	89.9	19.9

Abbreviation: Max, maximum; Min, minimum; SD, standard deviation.

## Data Availability

The datasets generated and analyzed during the current study are available from the corresponding authors on reasonable request.
